# Changes in the nasopharyngeal and oropharyngeal microbiota in pediatric obstructive sleep apnea before and after surgery: a prospective study

**DOI:** 10.1186/s12866-024-03230-7

**Published:** 2024-03-08

**Authors:** Lucheng Fang, Aikebaier Tuohuti, Wanyue Cai, Xiong Chen

**Affiliations:** 1 Department of Otorhinolaryngology, Head and Neck Surgery, Zhongnan Hospital of Wuhan University, Wuhan, Hubei China; 2 Sleep medicine centre, Zhongnan Hospital of Wuhan University, Wuhan, Hubei China

**Keywords:** Microbiome, Upper airway, Pediatric OSA, 16S rRNA sequencing, Biomarker

## Abstract

**Objective:**

To explore the changes and potential mechanisms of microbiome in different parts of the upper airway in the development of pediatric OSA and observe the impact of surgical intervention on oral microbiome for pediatric OSA.

**Methods:**

Before adeno-tonsillectomy, we collected throat swab samples from different parts of the oropharynx and nasopharynx of 30 OSA patients and 10 non-OSA patients and collected throat swab samples from the oropharynx of the above patients one month after the adeno-tonsillectomy. The 16 S rRNA V3–V4 region was sequenced to identify the microbial communities. The correlation analysis was conducted based on clinical characteristics.

**Results:**

There was a significant difference of alpha diversity in different parts of the upper airway of pediatric OSA, but this difference was not found in children with non-OSA. Beta diversity was significantly different between non-OSA and pediatric OSA. At the genus level, the composition of flora in different parts is different between non-OSA and pediatric OSA. The correlation analysis revealed that the relative abundance of *Neisseria* was significantly correlated with obstructive apnea hypopnea index. Furthermore, the functional prediction revealed that pathways related to cell proliferation and material metabolism were significantly different between non-OSA and pediatric OSA. Besides, the adeno-tonsillectomy has minimal impact on oral microbiota composition in short term.

**Conclusion:**

The changes in upper airway microbiome are highly associated with pediatric OSA. The relative abundance of some bacteria was significantly different between OSA and non-OSA. These bacteria have the potential to become new diagnostic and early warning biomarkers.

**Supplementary Information:**

The online version contains supplementary material available at 10.1186/s12866-024-03230-7.

## Introduction

Pediatric obstructive sleep apnea (OSA) is characterized by the recurrent incidence of either partial or complete obstruction of the upper airway during sleep, which disturbs the normal sleep quality and sleep architecture, leads to long-term sleep hypoxia and hypercapnia in children, and causes a series of pathophysiological changes, including growth retardation, behavioral cognitive impairment, memory and intellectual decline in children, mainly manifested as snoring, suffocation, mouth opening breathing with the high incidence rate among children(1.2%-5.7%) [1, 2]. To some extent, OSA is more harmful to children than adults. Hence, the timely detection and intervention of pediatric OSA holds substantial importance in improving the prognosis.

Different from OSA in adult, the main cause of upper airway obstruction in children is adenoid and/or tonsillar hypertrophy [3]. The adenoids and tonsils, as integral components of the immune system, exert a significant influence on the body’s immune response to infectious pathogens within the upper respiratory tract [4]. There are many folds and recesses on the surface of adenoids and tonsils, which are occupied by symbiotic microorganisms shortly after birth [5]. These microorganisms subsequently affect the development of innate mucosal immune response, which may be an important factor in protecting or inducing individuals to produce mucosal immune response during infection [5]. It is reported that most patients with chronic tonsillitis have a layer of “bacterial biofilm” attached to the surface of the tonsils [6]. This biofilm is formed by bacteria adhering to the surface of the body to produce various polysaccharide matrices, proteins, and other complex substances that aggregate with each other, causing various bacteria to adhere to form microbiota colonies, resulting in membrane like substances.

Prior research has established a significant association between the oral microbiota and the development of various systemic ailments, including cardiovascular disease, rheumatoid arthritis, and complications arising from HIV [7]. Presently, the investigation of the upper respiratory microbiota in pediatric patients with OSA primarily centers on the microbiota in the vicinity of the tonsils and adenoids. Certain scholars have posited the “pathogen reservoir hypothesis,” which proposes that the adenoids serve as the origin of pathogens in the tonsils and middle ear; however, additional substantiation of this hypothesis is required [8, 9]. Furthermore, the “bacterial interference theory” has been proposed by certain individuals, which posits that oral symbiotic bacteria impede the growth of pathogens by engaging in resource competition or generating antagonistic nutrients [10]. Recent investigation also demonstrated a significant correlation between adenoid hypertrophy and alterations in the microbiota composition of the upper airways compared to healthy subjects [11]. As a result, the alterations in upper airway microbiota may be closely related to the presence of pediatric OSA, and in-depth research on the correlation between upper airway microbiota and pediatric OSA has important reference value.

In this study, we collected swab samples from different parts of the participants’ nasopharynx and oropharynx. In addition, we also collected oral swab samples from participants after adeno-tonsillectomy. 16 S rRNA pyrosequencing was used to investigate the diversity of upper airway microbiome in pediatric OSA. The objective of this study is (1) to examine the microbial community characteristics of various regions within the upper airway in pediatric patients with OSA; (2) to study whether there are differences in the composition and diversity of microbial communities between pediatric patients with OSA and pediatric patients with non-OSA; (3) to observe the impact of adeno-tonsillectomy on the oral microbiome of pediatric patients with OSA.

## Methods

### Study participant

This study was designed and performed based on the Helsinki Declaration and the prospective specimen collection [12]. Ethical clearance was approved by the Ethical Review Board of the Zhongnan Hospital of Wuhan University (No.2,021,056). The written consent of every participant was obtained prior to the commencement of the study. All samples were collected exclusively from the Zhongnan Hospital of Wuhan University. Each participant exhibited symptoms of snoring and underwent standard polysomnography. All participants were diagnosed with adenoid and tonsillar hypertrophy through nasopharyngeal endoscopy. Severity of sleep apnea was categorized by Society of Otorhinolaryngology Head and Neck Surgery in Chinese Medical Association criteria [13]: non-OSA (obstructive apnea hypopnea index [OAHI], < 1 events/h), mild OSA (OAHI, 1–5 events/h), moderate OSA (OAHI, 6–10 events/h), and severe OSA (OAHI > 10 events/h). Participants were excluded if they met any of the following criteria: (1) There are infected lesions in the oral cavity; (2) People or their family members with acute respiratory infectious diseases; (3)People with nose, pharynx, throat diseases and chronic respiratory diseases that affect sleep; (4)Patients with dental caries and periodontal disease; (5)Abnormal preoperative biochemical examination results. The participants were categorized into four groups, namely the non-OSA group, mild OSA group, moderate OSA group, and severe OSA group, based on the results obtained from polysomnography.

### Sample collection and DNA extraction

**Fig. 1:**
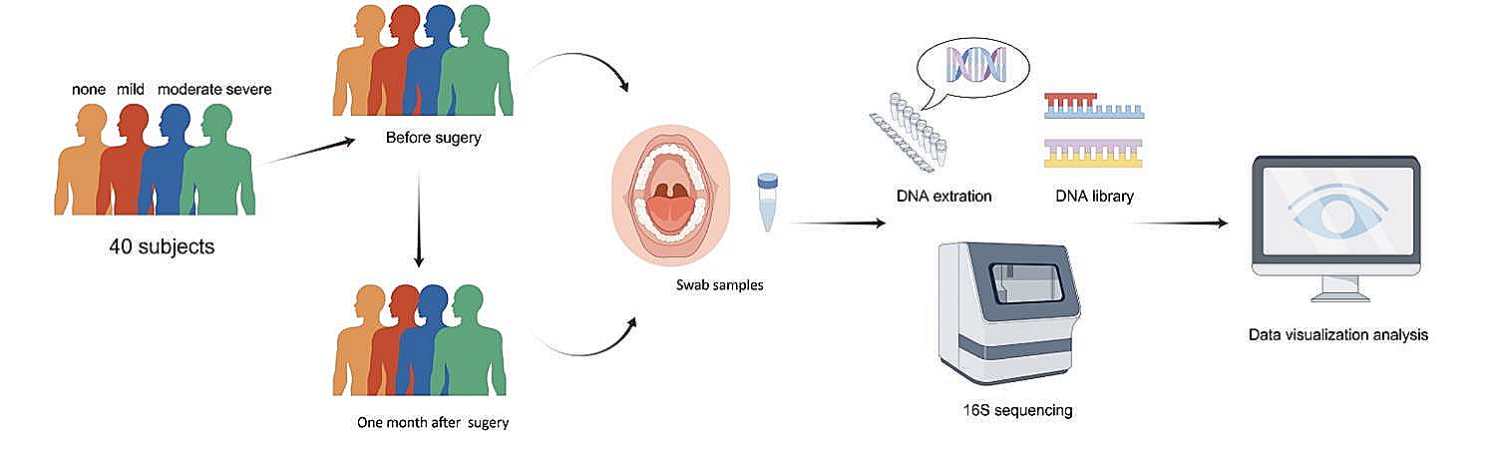
Overview of study design. Data analysis of the microbiome was performed to explore their correlation and relevance to clinical parameters

All subjects were forbidden to brush their teeth, rinse their mouth and eat the preceding night before collection. The trained professionals collected samples during adenotonsillectomy. Sterile throat swab shall be used to wipe the mucosa of the patient’s tongue base (oropharynx site)(Part A), surface of palatine tonsil (Part B), palatine tonsillar capsule after tonsillectomy (Part C) and adenoid (nasopharynx site) (Part D). The detailed collection location is shown in supplementary Figure S1. At the same time, it shall be noted that the throat swab shall not contact the mucosa of both sides of the mouth and cheek, hard palate teeth and other parts. Similarly, during a follow-up one month after adeno-tonsillectomy, swab samples from the participants’ tongue base were collected. After collection, put the sample into the liquid nitrogen tank for quick freezing and quickly transfer it to the laboratory (Fig. 1).

After placing swab samples in TENS buffer (5 M Sodium Chloride, 10% SDS, Triton X-1000, Tris-HCl, EDTA), 10% SDS and 20 mg/mL proteinase K, the samples were incubated overnight at 55°C. Proteins were eliminated through phenol/chloroform/isoamyl alcohol extractions, and DNA was subsequently precipitated using isopropanol on the following day. After being washed in 75% ethanol, the DNA was then resuspended in TE buffer. The quantification of DNA was conducted using the Qubit Fluorometer, employing the Qubit dsDNA BR Assay kit (Invitrogen, USA), while the quality assessment involved running an aliquot on a 1% agarose gel. The amplification of the variable regions V3–V4 of the bacterial 16S rRNA gene was achieved using degenerate PCR primers, namely 341F (5’-ACTCCTACGGGAGGCAGCAG-3’) and 806R (5’-GGACTACHVGGGTWTCTAAT-3’). Both the forward and reverse primers were modified with Illumina adapter, pad, and linker sequences. PCR enrichment was conducted in a 50 µL reaction mixture containing 30ng of template, fusion PCR primer, and PCR master mix. The PCR cycling conditions consisted of an initial denaturation step at 94 °C for 3 min, followed by 30 cycles of denaturation at 94 °C for 30 s, annealing at 56 °C for 45 s, extension at 72 °C for 45 s, and a final extension step at 72 °C for 10 min. The resulting PCR products were purified using AmpureXP beads and eluted in Elution buffer. The quality of the libraries was assessed using the Agilent 2100 bioanalyzer (Agilent, USA). The sequencing process on the Illumina MiSeq platform (BGI, Shenzhen, China) utilized validated libraries and adhered to the standard pipelines of Illumina, resulting in the production of 2 × 300 bp paired-end reads.

### Bacterial 16  rDNA sequencing and bioinformatic analysis

The raw reads underwent filtration to eliminate adaptors, low-quality, and ambiguous bases. Subsequently, the paired-end reads were merged with tags using the Fast Length Adjustment of Short reads program (FLASH, v1.2.11) [14] to obtain the tags. These tags were then clustered into operational taxonomic units (OTUs) using the UPARSE software (v7.0.1090) [15] with a 97% cutoff value. Additionally, chimera sequences were identified by comparing them with the Gold database using UCHIME (v4.2.40) [16]. Subsequently, the taxonomic classification of OTU representative sequences was conducted using the Ribosomal Database Project (RDP) Classifier v.2.2, employing a minimum confidence threshold of 0.6. The classifier was trained on the Greengenes database v201305 by QIIME v1.8.0 [17]. To obtain the OTU abundance statistics table for each sample, the USEARCH_global [18] was employed to compare all Tags back to OTU. At the OTU level, MOTHUR (v1.31.2) [19] and QIIME (v1.8.0) [17] were utilized to estimate alpha and beta diversity, respectively. Principal Coordinate Analysis (PCoA) was conducted using QIIME (v1.8.0) [17]. Sample clustering was performed by QIIME (v1.8.0) [15] using the UPGMA method. LEfSe clustering and LDA analysis were carried out using LEfSe (https://huttenhower.sph.harvard.edu/galaxy/). COG and KEGG functions were predicted using the PICRUSt software [20]. A barplot representing different classification levels was generated using GraphPad Prism (version 9.4.1). The raw data of this study were deposited in the Sequence Read Archive (https://www.ncbi.nlm.nih.gov/sra/) with the accession numbers PRJNA996776.

### Statistical analysis

The Mann-Whitney test was employed to assess the presence of a significant difference between the two groups in relation to continuous variables, while the Kruskal-Wallis test was utilized to evaluate the existence of a significant difference among multiple groups. In order to examine any disparities between groups in terms of frequency distribution, the chi-square test was employed. The dependent variables in this study were the clinical variables, while the independent variables consisted of the microbiota characteristics associated with OSA. Multiple linear regression analysis was conducted to examine the internal correlation between these variables. The statistical analysis was performed using GraphPad Prism (version 9.4.1), and *p* < 0.05 was considered statistically significant.

## Results

### Characteristics of the participants

This study included a cohort of 40 participants, comprising 10 individuals diagnosed with non-OSA, 10 individuals with mild OSA, 10 individuals with moderate OSA, and 10 individuals with severe OSA. The average age of the patients was 7.1 ± 2.9 years old, including 10 female and 30 male. Except for OAHI, micro-arousal index (MAI) and lowest blood oxygen saturation (LSaO2), there were no statistically significant differences observed among the four groups with regards to age, sex, BMI, mean blood oxygen saturation (MBOS), and white blood cell count (WBCC) (*P* > 0.05). The detailed demographic and clinical characteristics of the four groups are presented in Table 1. Following the screening process, 160 samples from 40 patients were subjected to 16 S rRNA MiSeq sequencing in accordance with established standards.

**Table 1:** Demographics and Clinical Characteristics

	Non-OSA	Mild OSA	Moderate OSA	Severe OSA	P-Value
N	10	10	10	10	
Age, yr	6.6(4–12)	6.6(4–10)	7.5(4–12)	8(3–13)	0.76
Sex					0.15
Female	2	2	4	2	
Male	8	8	6	8	
BMI, kg/m2	17.51	16.06	19.42	17.18	0.32
OAHI,events/h	0.48	2.5	7.2	23.9	< 0.001
LSaO_2_, %	90.3	88	84.3	83.2	< 0.001
MAI	8.52	10.25	12.27	16.69	0.018
MBOS, %	96.14	95.92	95.82	95.69	0.65
WBCC, /10^9	6.06	6.28	6.42	6.2	0.85

Abbreviations: BMI, Body mass index; OAHI, Obstructive apnea hypopnea index; LSaO2, lowest blood oxygen saturation; MAI, micro-arousal index; MBOS, mean blood oxygen saturation; WBCC, white blood cell count

### Microbiome diversity analysis

**Fig. 2:**
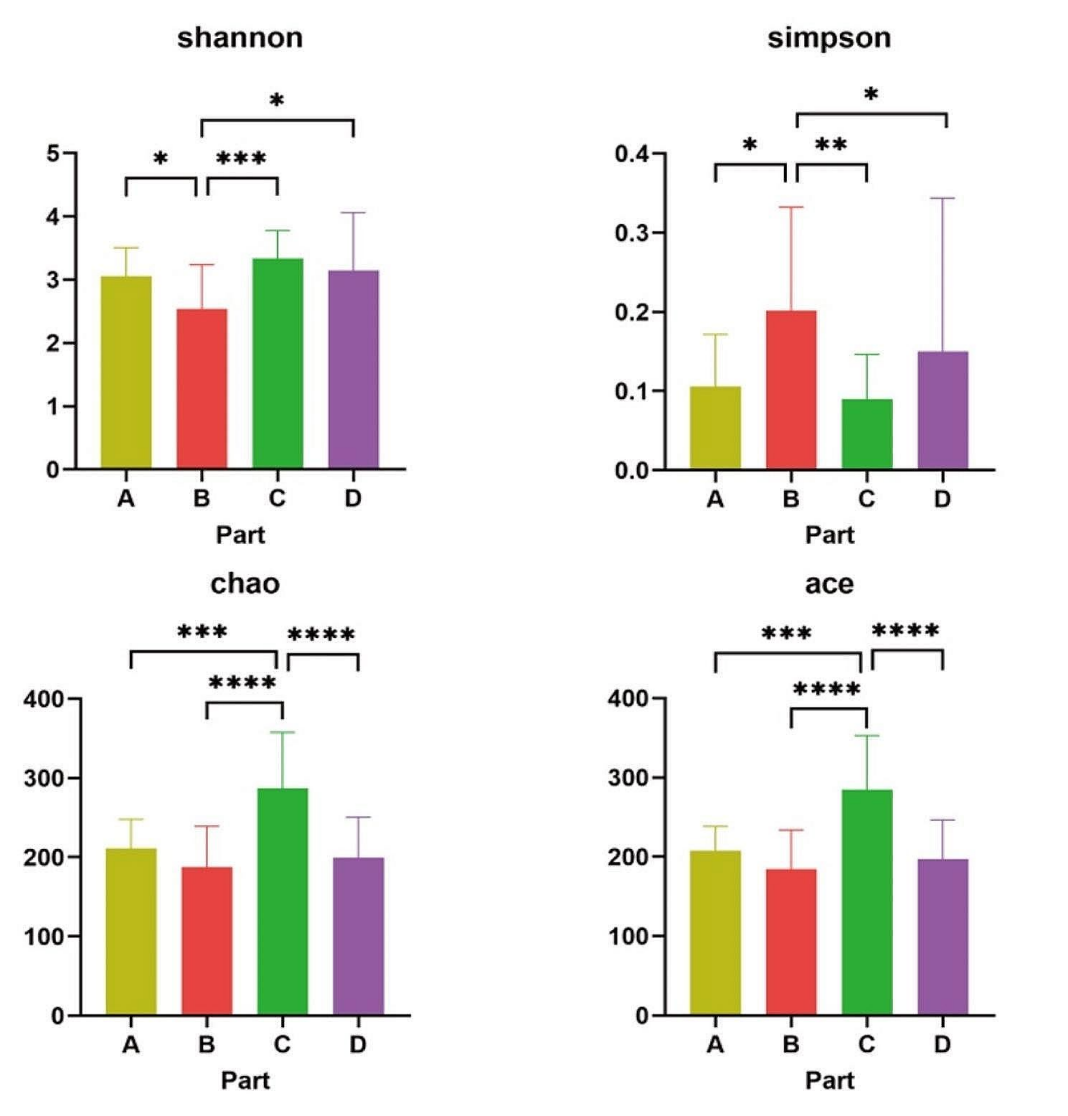
The microbial alpha diversity comparison among four parts in OSA group (*P < 0.05, **P < 0.01, ***P < 0.001, ****P < 0.0001)

The differences in microbiome between children without OSA and children with various severity of OSA were evaluated. Analysis of alpha diversity in OSA groups showed that part B had significantly decreased shannon index and significantly increased simpson index compared with other parts (Fig. 2), while part C had significantly increased chao index and ace index compared with other parts (Fig. S2). However, the statistically significant difference of alpha diversity index among four parts in non-OSA group was different from OSA groups (Figure S2). Among non-OSA group and various severity of OSA groups, alpha diversity had no statistically significant difference in same parts of upper airway(*P* > 0.05) (Figure S3).

**Fig. 3:**
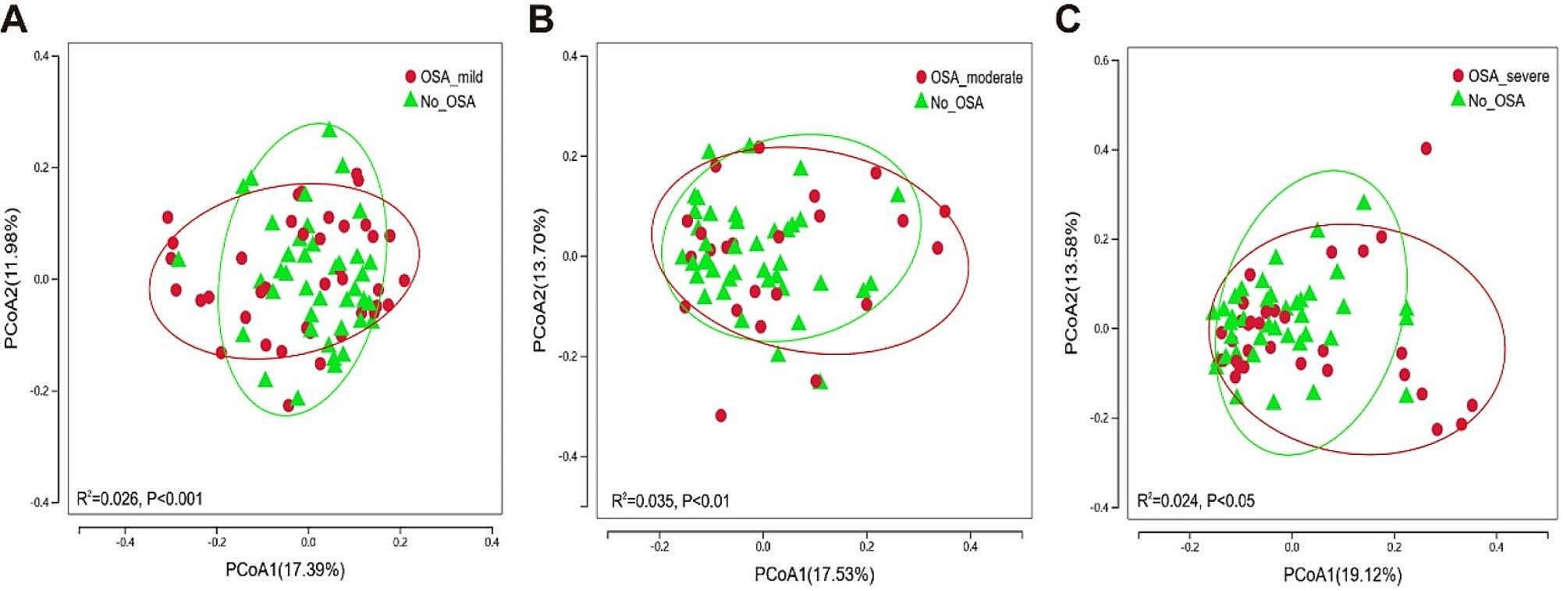
Comparisons of beta diversity by PCoA between non-OSA group and various severity of OSA group. (**A**) Mild OSA vs. Non-OSA (R^2^ = 0.026, *P* < 0.01); (**B**) Moderate OSA vs. Non-OSA (R^2^ = 0.035, *P* < 0.01); (**C**) Severe OSA vs. Non-OSA (R^2^ = 0.024, *P* < 0.05)

In order to visually represent the variation in microbiome composition across samples, beta diversity was assessed using Principal Coordinate Analysis (PCoA). PCoA of unweighted UniFrac (Fig. 3) showed that the samples of non-OSA group and various severity of OSA group were significantly separated, indicating that the beta diversity of overall upper airway microbial composition was different between non-OSA group and various severity of OSA group. On the contrary, there is no significant difference in beta diversity among various severity of OSA group (Figure S4).

### Composition and comparison of the Microbiome

**Fig. 4:**
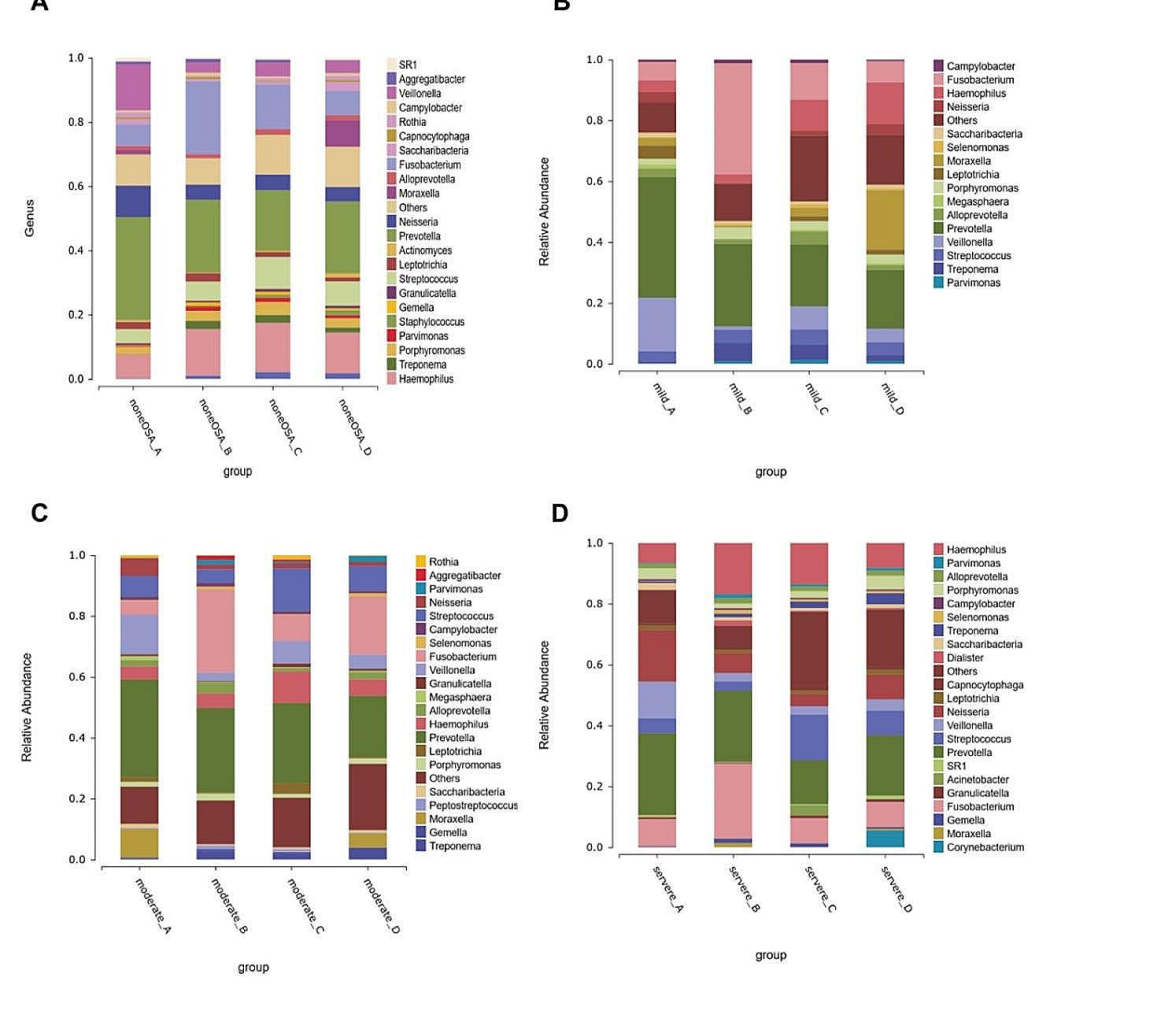
The microbial relative abundance at the genus level in four parts. (**A**) Non-OSA; (**B**) Mild OSA; (**C**) Moderate OSA; (**D**) Severe OSA

Based on the annotation of OTUs, we calculated the composition of microbiomes and plotted the microbial relative abundance on different levels in four parts of non-OSA group and various severity of OSA group. The composition of upper airway microbiome was different among different parts. At the genus level in part A, the proportion of *Prevotella* and *Veillonella* was up to 44-57% in non-OSA group, mild OSA group and moderate OSA group (Fig. 4), while in severe OSA group, the proportion of *Prevotella*, *Neisseria* and *Veillonella* was the highest (Fig. 4). At the genus level in part B, the proportion of *Prevotella* and *Fusobacterium* was up to 45-63% in non-OSA group and various severity of OSA group (Fig. 4). At the genus level in part C, the proportion of *Prevotella*, *Haemophilus* and *Fusobacterium* were the highest in non-OSA group and mild OSA group (Fig. 4), and the proportion of *Prevotella*, *Haemophilus* and *Streptococcus* were the highest in moderate OSA group and severe OSA group (Fig. 4). At the genus level in part D, the proportion of *Prevotella* and *Haemophilus* were the highest in non-OSA group, and the proportion of *Prevotella*, *Moraxella* and *Haemophilus* were the highest in mild OSA group, and the proportion of *Prevotella* and *Fusobacterium* were the highest in moderate OSA group, and the proportion of *Prevotella* and Others were the highest in severe OSA group (Fig. 4).

**Fig. 5:**
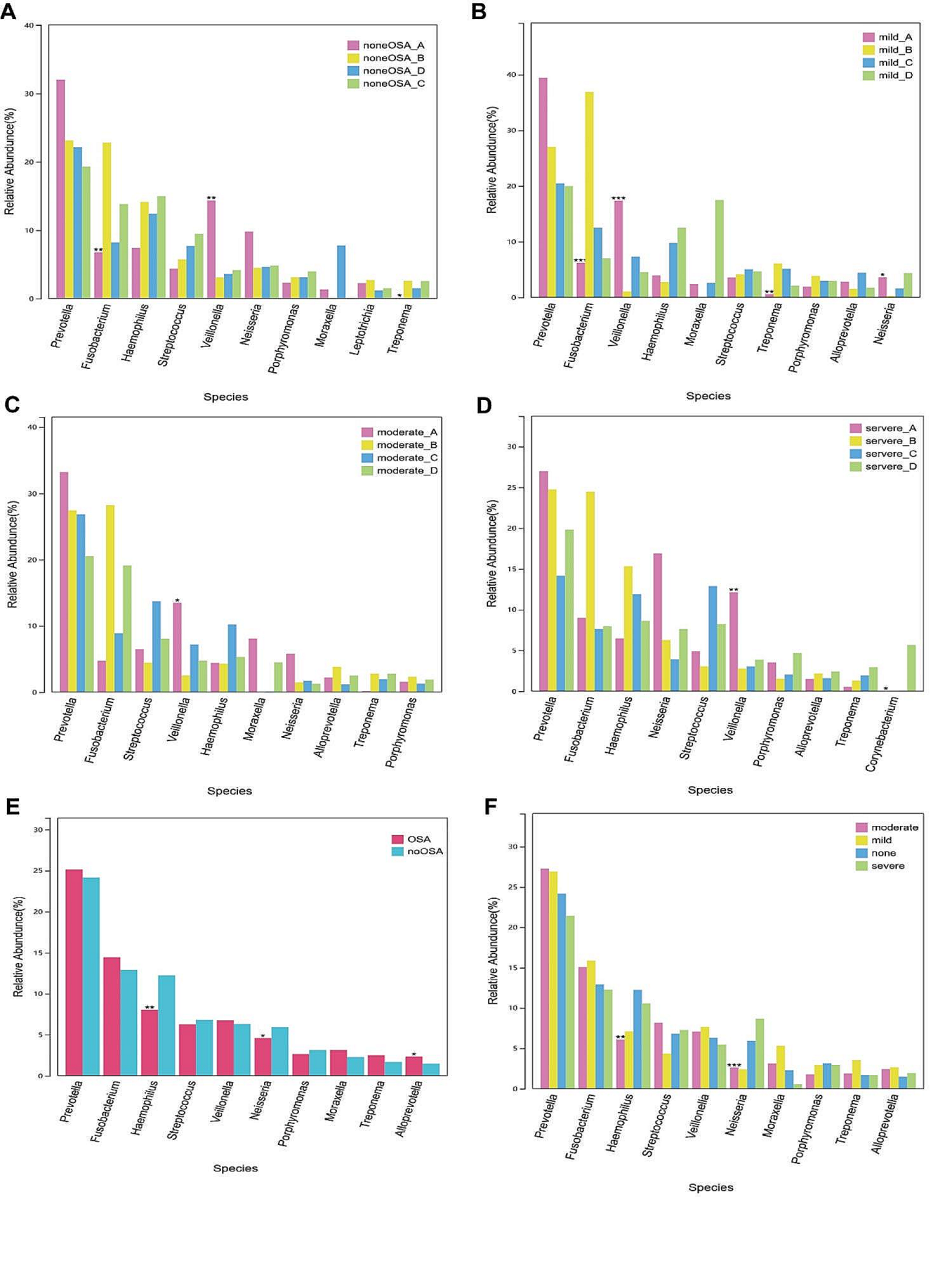
Comparison of key species differences.(**A**) Comparison of four parts in non-OSA group; (**B**) Comparison of four parts in mild OSA group; (**C**) Comparison of four parts in moderate OSA group; (**D**) Comparison of four parts in severe OSA group; (**E**) OSA vs. Non-OSA; (**F**) Non-OSA vs. mild OSA vs. moderate OSA vs. severe OSA (*P < 0.05, **P < 0.01, ***P < 0.001)

At the genus level, we selected the 10 species with the highest abundance to compare the relative abundance differences of each species in each group. In non-OSA group, the relative abundance of *Fusobacterium*, *Veillonella* and *Treponema* were significantly different among the four parts (Fig. 5A). In mild OSA group, the relative abundance of *Fusobacterium*, *Veillonella, Treponema* and *Neisseria* were significantly different among the four parts (Fig. 5B). In moderate OSA group, the relative abundance of *Veillonella* was significantly different among the four parts (Fig. 5C). In severe OSA group, the relative abundance of *Veillonella* and *Corynebacterium* were significantly different among the four parts (Fig. 5D). Overall, the relative abundance of *Haemophilus*, *Neisseria* and *Alloprevotella* were significantly different between OSA group and non-OSA group (Fig. 5E), and the relative abundance of *Haemophilus* and *Neisseria* were significantly different between non-OSA group and various severity of OSA group (Fig. 5F). In part A and part D, there was no significant difference in the relative abundance of bacterium between non-OSA group and various severity of OSA group (Figure S5). In part B, the relative abundance of *Haemophilus*, *Treponema* and *Neisseria* were significantly different between non-OSA group and various severity of OSA group (Figure S5). In part C, the relative abundance of *Prevotella* was significantly different between non-OSA group and various severity of OSA group (Figure S5).

### Phylogenetic characteristics of microbial communities

Using Lefse analysis and linear discriminant analysis (LDA) Score, we conducted a characterization of the upper airway microbiota in both the non-OSA group and the various severity levels of the OSA group. The phylogenetic distribution of upper airway microbiomes associated with non-OSA and the different severity levels of OSA is visually represented in the cladogram (Figure S6). The histograms depicting the LDA scores were computed, revealing significant difference in bacterial composition between the non-OSA group and the different severity levels of the OSA group (Figure S7). In Figure S7A, 22 kinds of biomarkers were significantly enriched in non-OSA group (*P* < 0.05; LDA score > 2), and 25 kinds of biomarkers were significantly enriched in mild OSA group (*P* < 0.05; LDA score > 2). In Figure S7B, 13 kinds of biomarkers were significantly enriched in non-OSA group (*P* < 0.05; LDA score > 2), and 16 kinds of biomarkers were significantly enriched in moderate OSA group (*P* < 0.05; LDA score > 2). In Figure S7C, 5 kinds of biomarkers were significantly enriched in non-OSA group (*P* < 0.05; LDA score > 2), and 30 kinds of biomarkers were significantly enriched in severe OSA group (*P* < 0.05; LDA score > 2).

### Multivariate analysis for microbiota signatures

The clinical variables (Age, BMI, OAHI, MAI, MBOS and WBCC) were taken as dependent variables respectively, and the relative abundance of the different microflora was taken as independent variables. We mainly analyzed the correlation between dependent variable and independent variable in the tonsil area (Tonsil recess and Tonsillar capsule after tonsillectomy). As shown in Table S1, the relative abundance of various microflora was significantly correlated with clinical characteristics. However, only the relative abundance of *Neisseria* was significantly correlated with OAHI (*P* < 0.05).

### Function analysis

To investigate the potential mechanisms underlying the upper airway microbiome in pediatric OSA, we conducted an analysis utilizing Clusters of Orthologous Groups (COG) and Kyoto Encyclopedia of Genes and Genomes (KEGG). There were 25 functional pathwaysIn in COG analysis (Figure S8A) and 28 functional pathways in KEGG analysis (Figure S8B). What’s more, we compared the functional pathways with significant difference between non-OSA group and various severity of OSA group. In COG analysis, differential functional pathways between non-OSA group and various severity of OSA group were mainly related to cellular processes and information storage. In KEGG analysis, differential functional pathways between non-OSA group and various severity of OSA group were mainly related to metabolism. The detailed information of functional pathways with significant differences were showed in Table S2.

### Postoperative oral microbiota characteristics

**Fig. 6:**
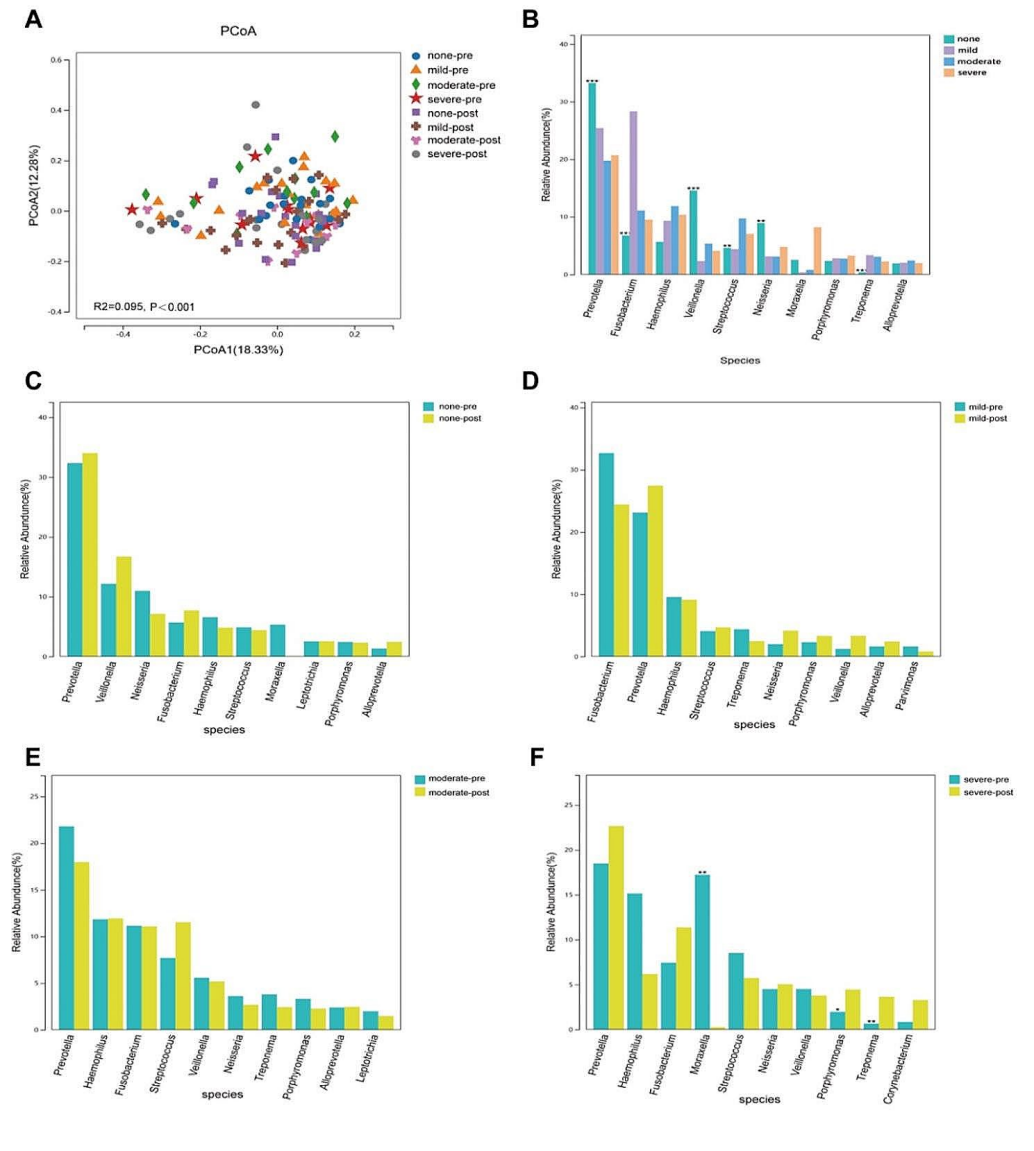
Postoperative oral microbiota characteristics. (**A**) Comparisons of beta diversity by PCoA among preoperative and postoperative groups; (**B**) Analysis of genus abundance of predominant bacteria among postoperative groups; (**C-F**) Analysis of genus abundance of predominant bacteria between preoperative and postoperative groups; C) non-OSA; (**D**) Mild OSA; (**E**) Moderate OSA; (**F**) Severe OSA

A total of 38 patients completed the collection of oral microbiota samples in tongue base (oropharynx site) after surgery. One month after the patient underwent adeno-tonsillectomy, there was no difference in alpha diversity of microbiota in the tongue base of each group (*P*>0.05), but the difference in beta diversity of microbiota still existed among the groups (*P*<0.001) (Figure S9). Comparison of preoperative and postoperative results, there was no difference in alpha diversity of oral microbiota (*P*>0.05) (Figure S10), while there was a difference in beta diversity of oral microbiota (*P*<0.001) (Fig. 6A). To evaluate the differences more intuitively between groups, we next conducted an analysis of genus abundance of predominant bacterial. The differential microbiota among the postoperative groups were Fusobacterium, *Veillonella*, *Streptococcus*, *Neisseria*, and *Treponema* (Fig. 6B). In addition, there was no significant difference in the relative abundance of predominant bacteria between the preoperative and postoperative groups in the non OSA group, mild OSA group and moderate OSA group (Fig. 6C-E). Only in the severe OSA group, there was a significant difference in the relative abundance of *Moraxella*, *Treponema*, and *Porphyromonas* in the predominant oral microbiota between preoperative and postoperative severe OSA patients (Fig. 6F).

## Discussion

Pediatric OSA is characterized by a range of pathophysiological alterations resulting from the recurrent incidence of partial or complete obstruction in the upper airway during sleep, thereby disrupting the respiratory function and sleep patterns in children [2]. OSA is the most serious disease in pediatric sleep disordered breathing. Due to its elevated prevalence and consequential long-term complications, OSA has garnered increasing attention from both parents and society [1, 2]. The oral cavity is an important part of the upper respiratory tract of the human body. It stores a large number of biological information substances, such as human oral microorganisms, DNA, RNA, proteins, metabolites, etc., which can reflect the physiological and pathological conditions of the body locally and generally [21, 22]. Compared with blood and samples from other parts of the body, the collection of oral swabs has the advantages of non-invasive, convenient collection, convenient storage and transportation, and is conducive to large-scale screening of certain diseases [21]. The research on oral biomarkers has attracted more and more attention, and has been widely used to study the pathogenesis, drug monitoring and efficacy evaluation of various diseases, such as oral diseases (caries, periodontal disease, oral squamous cell carcinoma, etc.) [23, 24] and systemic diseases (autoimmune diseases, cardiovascular diseases, HIV, etc.) [25–27]. It is the complex interaction between the microbial flora and the host microenvironment that maintains the dynamic balance of the microecology of the upper airways. Therefore, studying the changes in the microbiota of the upper airwaysis is crucial in gaining a better comprehension of the pathogenesis of pediatric OSA.

Based on our research findings, a notable variation in alpha diversity was observed across distinct regions of the upper airway among children diagnosed with OSA, especially around the tonsils, but this difference was not found in children with non-OSA. In addition, the PCoA of upper airway microbiome can significantly distinguish patients with non-OSA and patients with OSA of different severity. The aforementioned findings suggested that there may be alterations in the upper airway microbiome of children diagnosed with OSA. In OSA patients, the fluctuation in upper airway pressure would significantly reduce airflow, which affects the moisture and oxygen concentration in the upper respiratory tract and could probably lead to disorders of the upper airway microbiome in patients with OSA [28].

In the previous study, Jervis et al. [29] analyzed the middle ear effusion, nasopharyngeal swab and adenoid biopsy samples of 11 children with secretory otitis media in Australia, and found that there were significant differences between the middle ear effusion, nasopharyngeal swab and adenoid flora, and *Haemophilus*, *Streptococcus* and *Moraxella* existed in all samples. Ren et al. [10] analyzed the microflora in adenoids of 67 children who had undergone surgical removal of adenoids, and found that the microflora of adenoids were very different from that of other parts of the human body. The microflora of adenoids was also different among different individuals, but the core flora was similar, mainly including four phyla: *Firmicutes*, *Proteobacteria*, *Fusobacteria* and *Actinobacteria*. Similarly, our study also found that the microbial composition of different parts was different. *Prevotella* was highly enriched in each part, while *Corynebacterium* was highly enriched only in the region of nasopharynx site of severe OSA. In addition, the relative abundance of *Prevotella*, *Fusobacterium*, *Veillonella*, *Treponema*, *Neisseria* and *Alloprevotella* between OSA group and non-OSA group was significantly different, which indicated that the flora of upper airway is diverse, and the oral-colonized bacteria in different parts are quite different. Pediatric OSA probably is the result of multiple symbiotic or pathogenic bacteria and their immune responses, rather than a single bacterium on the surface of tissues [30]. These symbiotic bacteria exist in the oral tissues of OSA patients for a long time, which may be the reason for the previous failure of antibiotic treatment for chronic tonsillitis. It also shows that our understanding of the pathogenesis of OSA in children is not deep enough.

Currently, there is no study on the change of upper airway microbiome with the development of OSA and the upper airway microbiome reflecting the severity of symptoms is also unclear. A previous large-scale study on nasal microbiome of adult OSA demonstrated that there was a significant and positive correlation observed between the abundance of Streptococcus in the nasal mucosa and the apnea-hypnea index in adult individuals with OSA [31]. The study by Marazzato et al. suggested that adenoid hypertrophy is correlated with alterations in the nasal microbiota composition when compared to individuals in a healthy state [11]. Given that there may be involvement of multiple bacteria in the occurrence and progression of pediatric OSA, we conducted a correlation analysis with the relative abundance of significantly different bacteria between groups as the independent variable and clinical characteristics as the dependent variable. It was found that in the tonsil area (Tonsil recess and tonsillar capsule), only the relative abundance of *Neisseria* had a significant correlation with OAHI. With the increase of OAHI, the relative abundance of *Neisseria* also increased.

*Neisseria* is a group of gram-negative diplococci with oxidase positive. *Neisseria gonorrhoeae* and *Neisseria meningitidis* in *Neisseria* are common pathogenic *Neisseria* causing human diseases, while other strains belong to normal flora or conditional pathogens parasitizing human upper airways [32]. According to report, certain strains of *Neisseria* that are not *gonococci* or *meningococcal* have the potential to cause primary or secondary respiratory tract infections, thereby posing a significant challenge to accurate clinical and laboratory diagnosis and treatment [33]. Additionally, it is noteworthy that symbiotic *Neisseria* exhibits immunosuppressive properties that can enhance the expression of host immunosuppressive molecules and impede the release of immune inducible factors into the host environment [34, 35]. The research conducted by Zhu et al. demonstrates that symbiotic *Neisseria* can prompt dendritic cells to generate immunosuppressive IL-10, which consequently restrains the proliferation of T cells upon antigenic stimulation [36]. It is reported that the bacteria that cause tonsillitis not only reside on the surface of the tonsils, but also in the deep tissues of the tonsils. Therefore, the culture results on the surface of the tonsils may not always indicate the true pathogen, leading to sometimes unsuccessful antibiotic treatment in clinical practice [37]. Consequently, it is reasonable to postulate that the proliferation of *Neisseria* within the tonsil area of pediatric patients with OSA may be intricately associated with the hypertrophy of tonsils. Nevertheless, the causative association between these factors remains uncertain and warrants further investigation for validation.

In KEGG analysis, we found that, compared with non OSA group, “Phosphonate and phosphinate metabolism”, “Tyrosine metabolism”, “Glutathione metabolism” and “Tryptophan metabolism” significantly increased in severe OSA group. This result is different from that of zhang et al., which indicated that the function of adenoid flora in pediatric OSA patients was characterized by decreased amino acid metabolism [38]. Previous study demonstrated that Nitrotyrosine levels were positively correlated with hypoxemia severity in OSA, and intracellular Glutathione level significantly increased for OSA patients [39, 40]. What’s more, some people hypothesized the increase in Tryptophan metabolism probably is important in OSA-related cardiovascular diseases and might be one of the players in the relationship between cancers and OSA [41]. All the above results indicate that the research on upper airway microbiome should not only focus on the change of microbial composition, but also explore the metabolic pathway of upper airway microecological function genes to enable people to better understand the pathogenesis of OSA.

To facilitate a more comprehensive analysis of the impact of surgical interventions on the oral microbiome of pediatric patients with OSA, oral swab samples were collected one month post adeno-tonsillectomy. Prior research has identified substantial alterations in the diversity of gut microbiome among patients following interventions such as surgical procedures or medication administration [42, 43]. What is more, Lin et al. reported that oral microbial diversity were significantly decreased in postoperative thyroid cancer patients [44]. However, in our analysis results, unexpectedly, there was no significant alterations between preoperative and postoperative groups in alpha diversity and relative abundance of predominant oral microflora, except for those with severe OSA. These findings indicate that while surgery may enhance the ventilatory function, it may not significantly affect the homeostasis of the upper airway microenvironment in short term. A prior randomized controlled trial demonstrated that the utilization of antibiotics at varying follow-up intervals resulted in significant alterations to the intestinal microbiota of the subjects, while the composition of oral microbiome remained relatively stable [45]. This suggests that oral microbiome may possess robust tolerance and resistance to numerous unfavorable conditions, which may partially elucidate the limited fluctuations in oral microbiome after the operation [46]. Additionally, it may also be caused by the transfer of bacterial communities in other parts of the upper airway. Therefore, the enduring influence of oral microbiota on pediatric patients with OSA may potentially contribute to the recurrence of adenoidectomy in certain children. Nonetheless, our findings can only serve as a point of reference, given the dearth of pertinent research on the impact of adeno-tonsillectomy on the microbiome of the upper airways.

Although we try to avoid the design defects of previous studies, our research still has some limitations. Firstly, 16 S rDNA sequencing rather than metagenome sequencing limits the further interpretation of species precision and function analysis in this study. Secondly, although strict inclusion and exclusion criteria were set, individual differences were inevitable, and there were always confounding factors such as genetics, diet, environment, etc. Third, the subjects only included children with OSA or non-OSA. It is necessary to further evaluate the impact of upper airway microbiome on adult OSA and include healthy people as controls. Fourth, this was a single center study. Most of the samples were obtained from nearby regions, and the sample size was comparatively limited. Consequently, it is imperative to conduct a multi-center study encompassing a substantial sample size to corroborate these findings. Finally, this study only provided evidence of correlation rather than causality. It is necessary to further experiment to verify whether candidate bacteria and their functional pathways related to pediatric OSA. Despite these limitations, our study offers valuable insights for future research exploring floral disturbances in the upper airway of pediatric patients with OSA.

## Conclusion

In summary, our study demonstrated that changes in upper airway microbiota, especially in the tonsil area, are highly associated with pediatric OSA. The relative abundance of some bacteria were significantly different among OSA groups and control group. The impact of adeno-tonsillectomy on oral microbiota composition is relatively small in the short term. The associations between upper airway microbiota and OSA require further investigations to evaluate causal relationships.

### Electronic supplementary material

Below is the link to the electronic supplementary material.

Supplementary Material 1

## Data Availability

Raw data in this study have been deposited into the Sequence Read Archive (SRA) and are publicly available under the BioProject accession number of PRJNA100021.
