# Supplementary material for: Changes in the nasopharyngeal and oropharyngeal microbiota in pediatric obstructive sleep apnea before and after surgery: a prospective study
Source: BMC Microbiol. 2024 Mar 8;24:79. doi: 10.1186/s12866-024-03230-7 (PMC10921815; doi:10.1186/s12866-024-03230-7)
Supplement: Supplementary file 1 — Supplementary Material 1 [file 12866_2024_3230_MOESM1_ESM.docx]

**Figure S1：**Graphic representation of sampling from four upper airway sites in each subject.

**Figure S2：**The microbial alpha diversity comparison among four parts in no OSA group. A: Tongue base; B: Surface of palatine tonsil; C: Palatine tonsillar capsule after tonsillectomy; D: Adenoid (ns: no significance, *P<0.05, **P<0.01).

**Figure S3：**The microbial alpha diversity comparison among four groups in four parts. A: Tongue base; B: Surface of palatine tonsil; C: Palatine tonsillar capsule after tonsillectomy; D: Adenoid (ns: no significance, *P<0.05, **P<0.01).

**Figure S4：**Comparisons of beta diversity by PCoA between various severity of OSA group.

**Figure S5：** The differences in the relative abundance of key bacterias among four groups in four parts. A: Tongue base; B: Surface of palatine tonsil; C: Palatine tonsillar capsule after tonsillectomy; D: Adenoid (ns: no significance, *P<0.05, **P<0.01).

**Figure S6:** The phylogenetic tree diagram by the LEfSe method showed the phylogenetic distribution of the upper airway microbiome related to four groups. The circles radiating from the inside to the outside represented the classification level from the phylum to the genus. Each circle at levels represented a classification at that level, and the diameter of the circle represents its relative abundance. The uniform coloring with no significant difference was yellow, and the biomarker with significant difference followed the grouping color for coloring. Species with an LDA score greater than 2 and a P value less than 0.05 were considered different species.

**Figure S7：**LEfSe and LDA analysis based on OTUs characterize the microbiome among four groups. Species with an LDA score greater than 2 and a P value less than 0.05 were considered different species.

**Figure S8：**The bacterial functions in four groups.

**Figure S9：**The microbial diversity comparison in tongue base site among four groups. A,B,C,D:

Alpha diversity index. E: Principal coordinates analysis plot based on the unweighted UniFrac

distance.

**Figure S10：**The microbial alpha diversity comparison between preoperative and postoperative groups. A: none OSA group; B: mild OSA group; C: moderate OSA group; D: severe OSA group (ns: no significance, *P<0.05, **P<0.01).

**Table S1**. Multivariate linear regression analysis between clinical characteristics and microbiota signatures in the tonsil area.

**Table S2.** Functional pathway difference analysis.

Figure S1
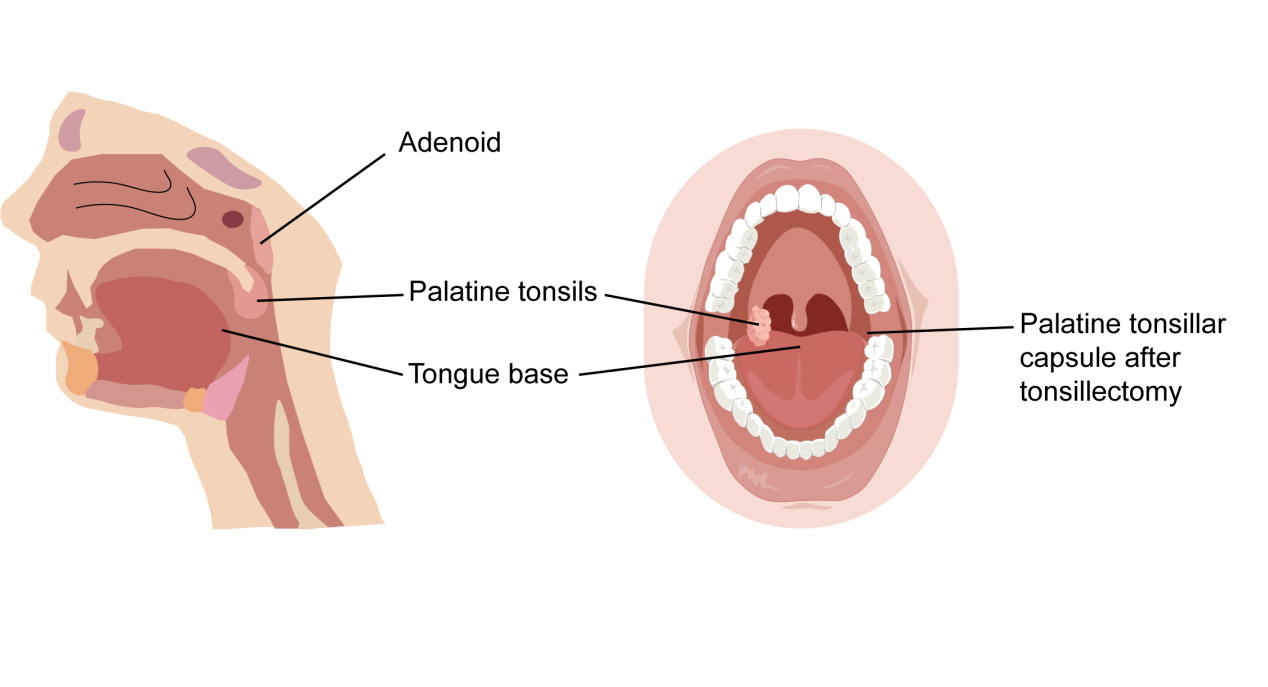


Figure S2


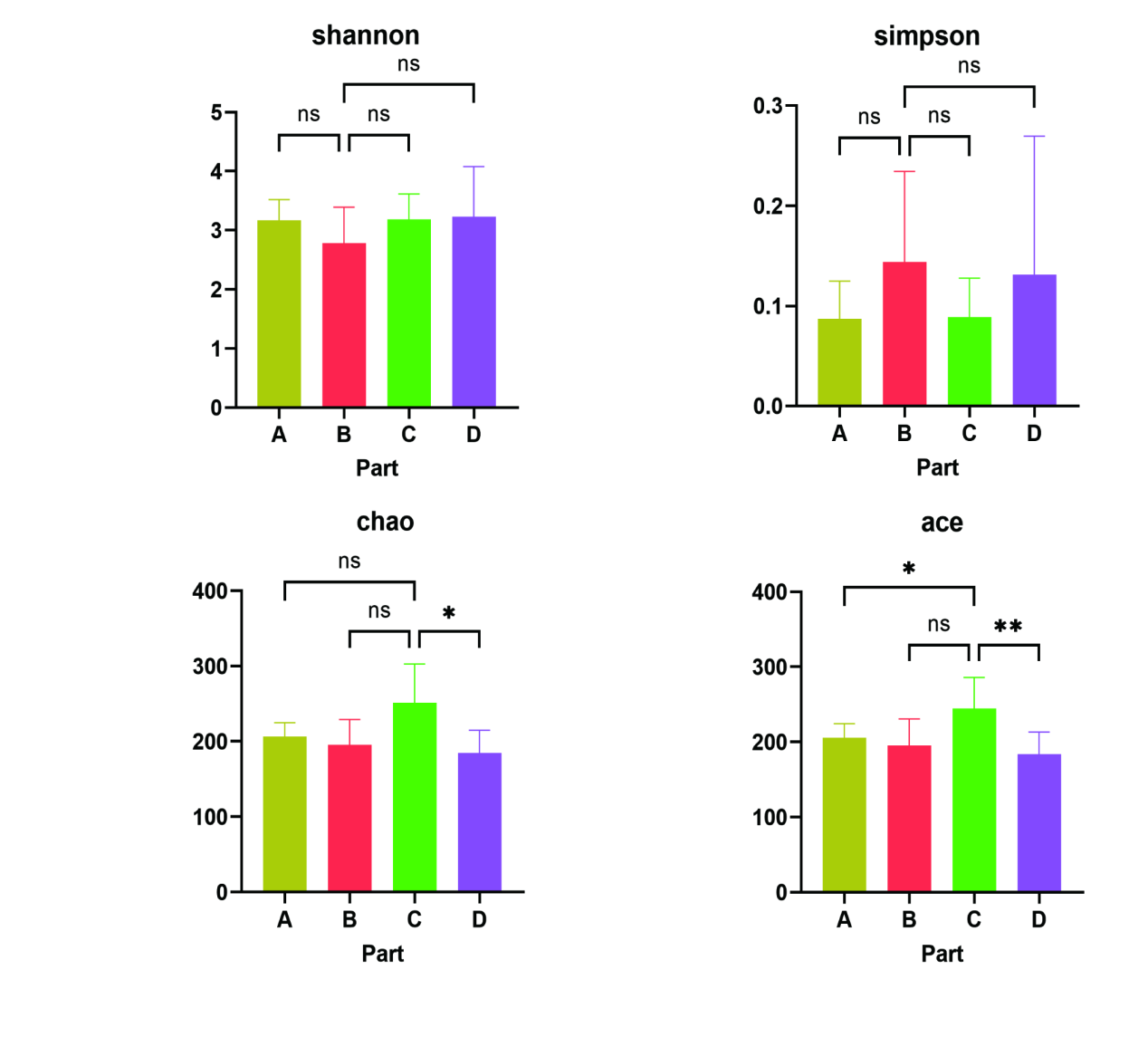


Figure S3


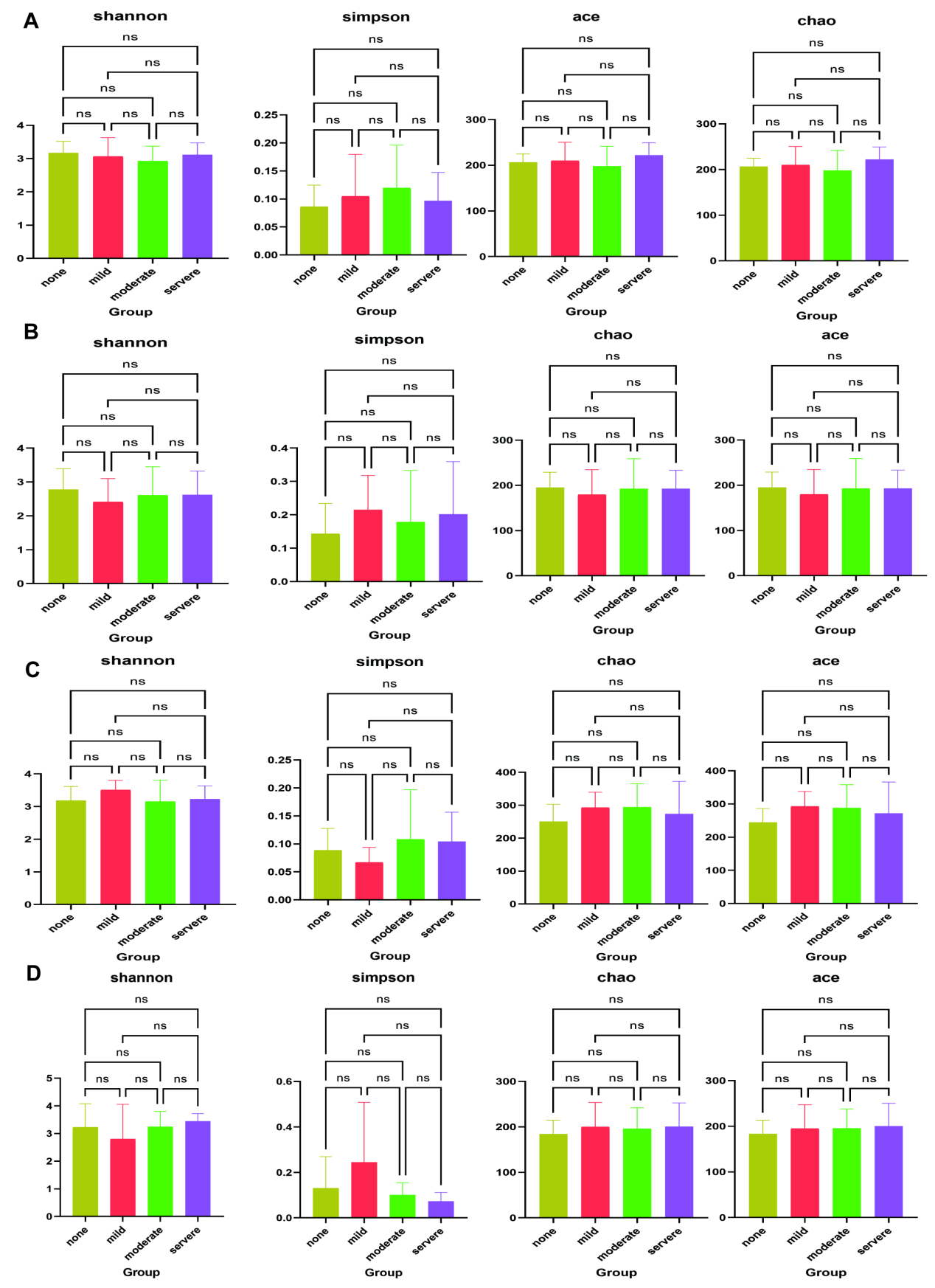


Figure S4


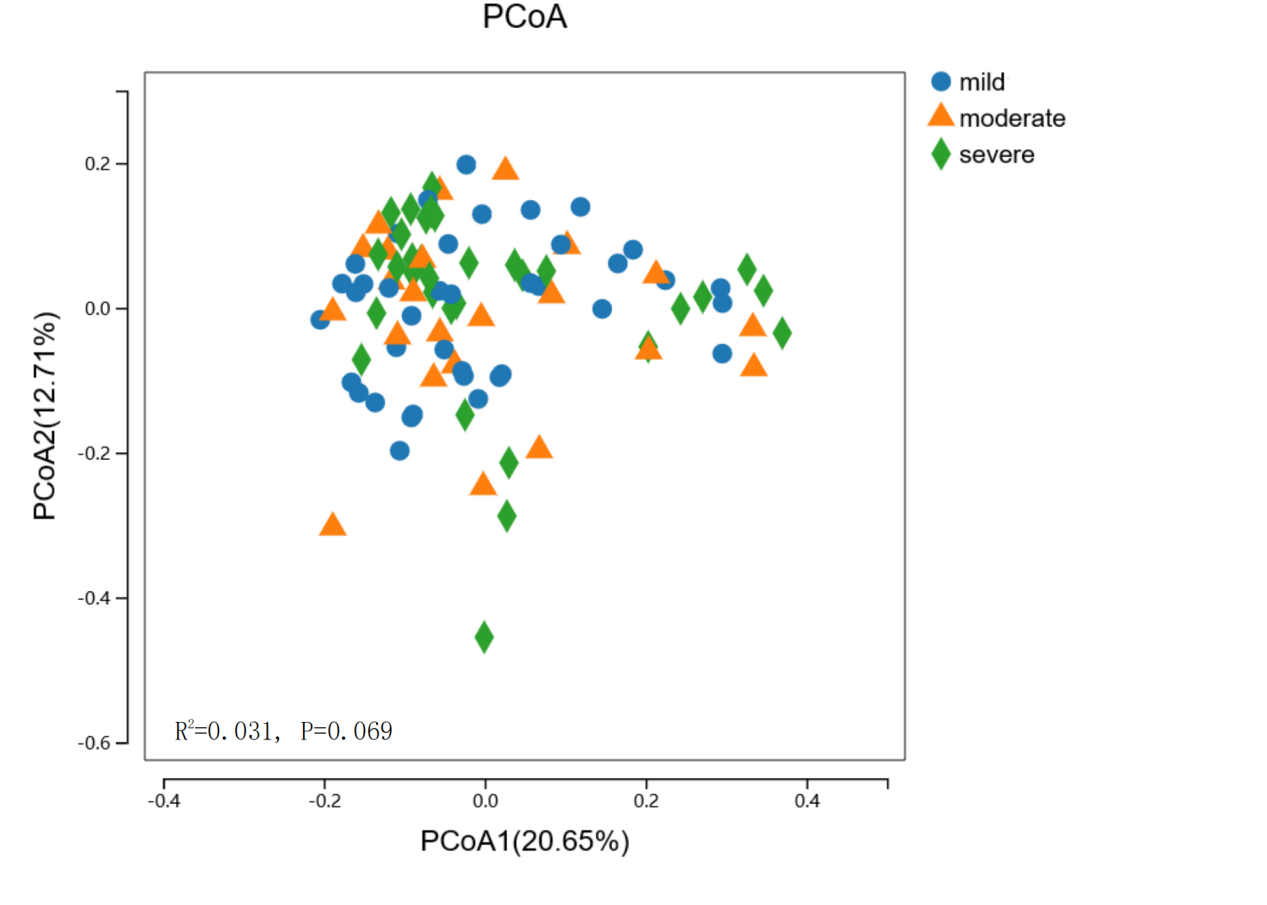


Figure S5


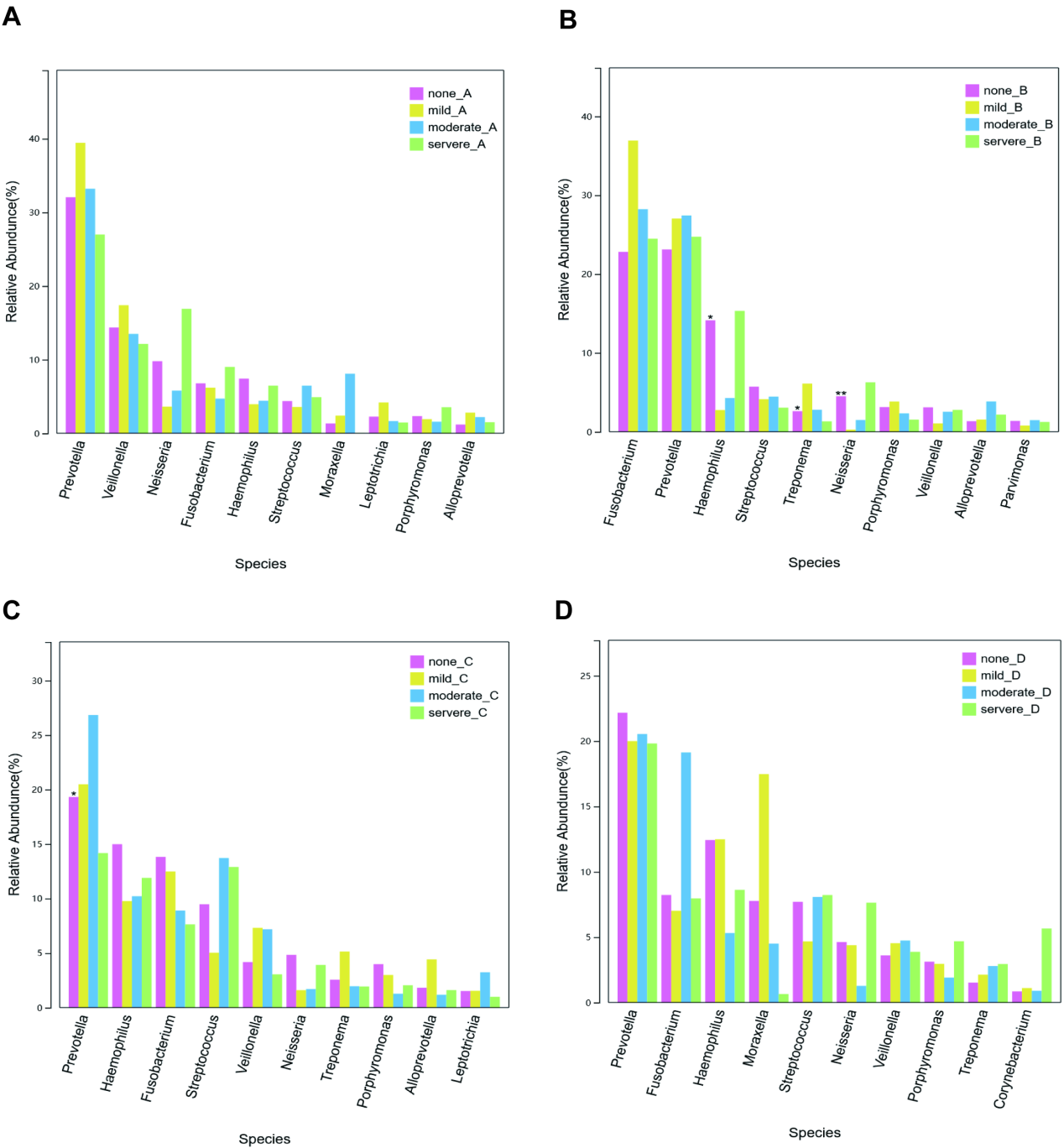


Figure S6


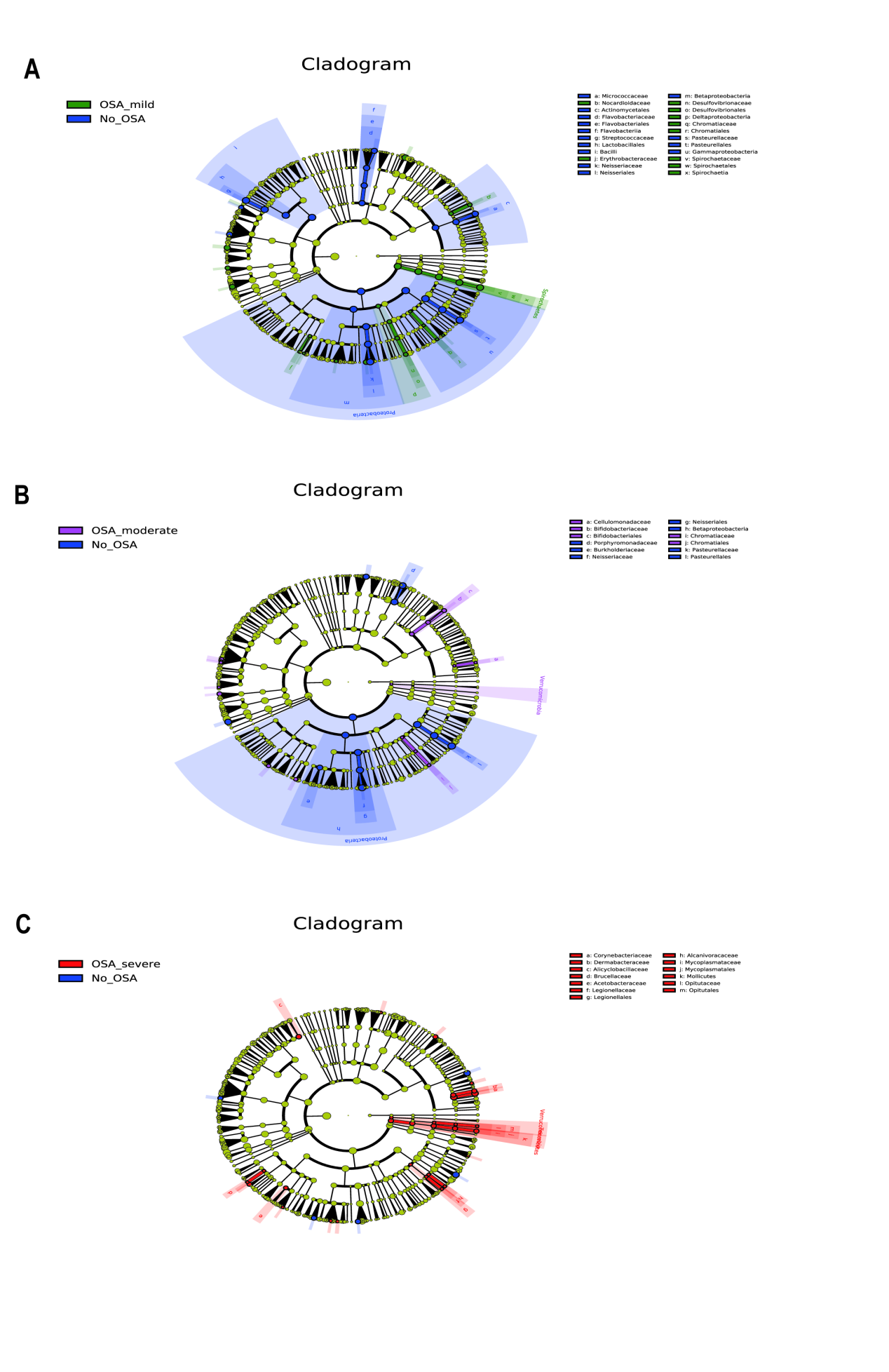


Figure S
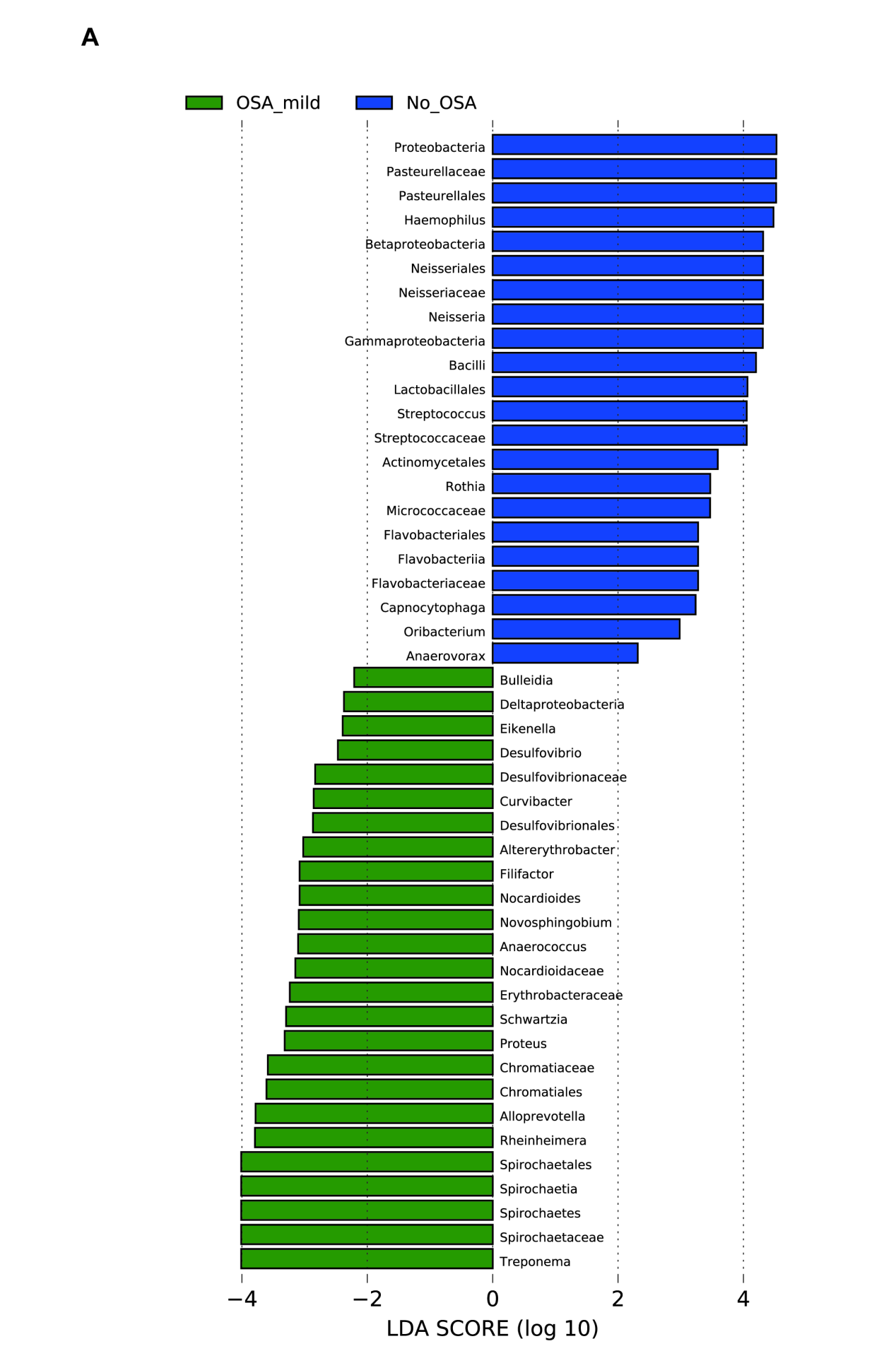
7


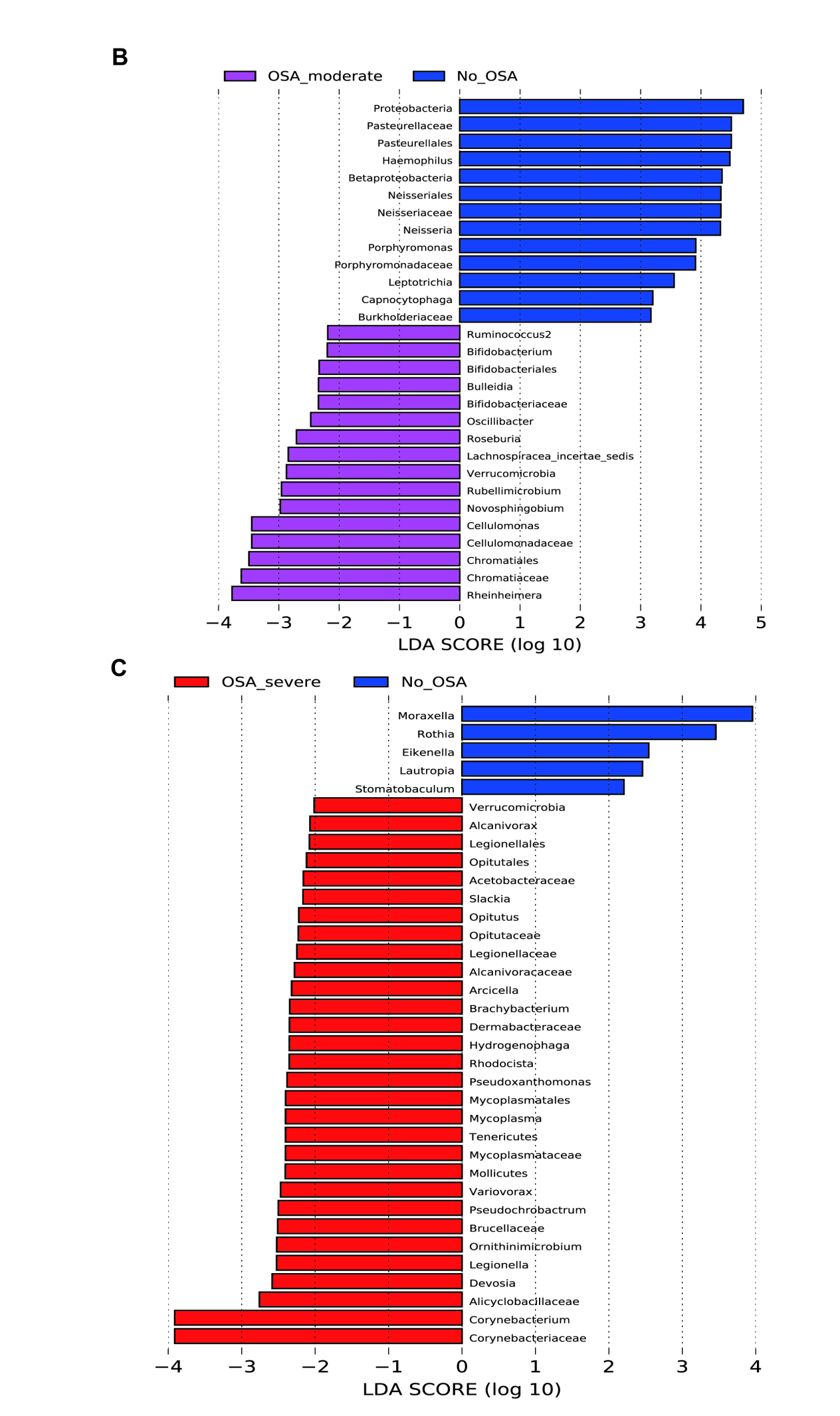


Figure S8


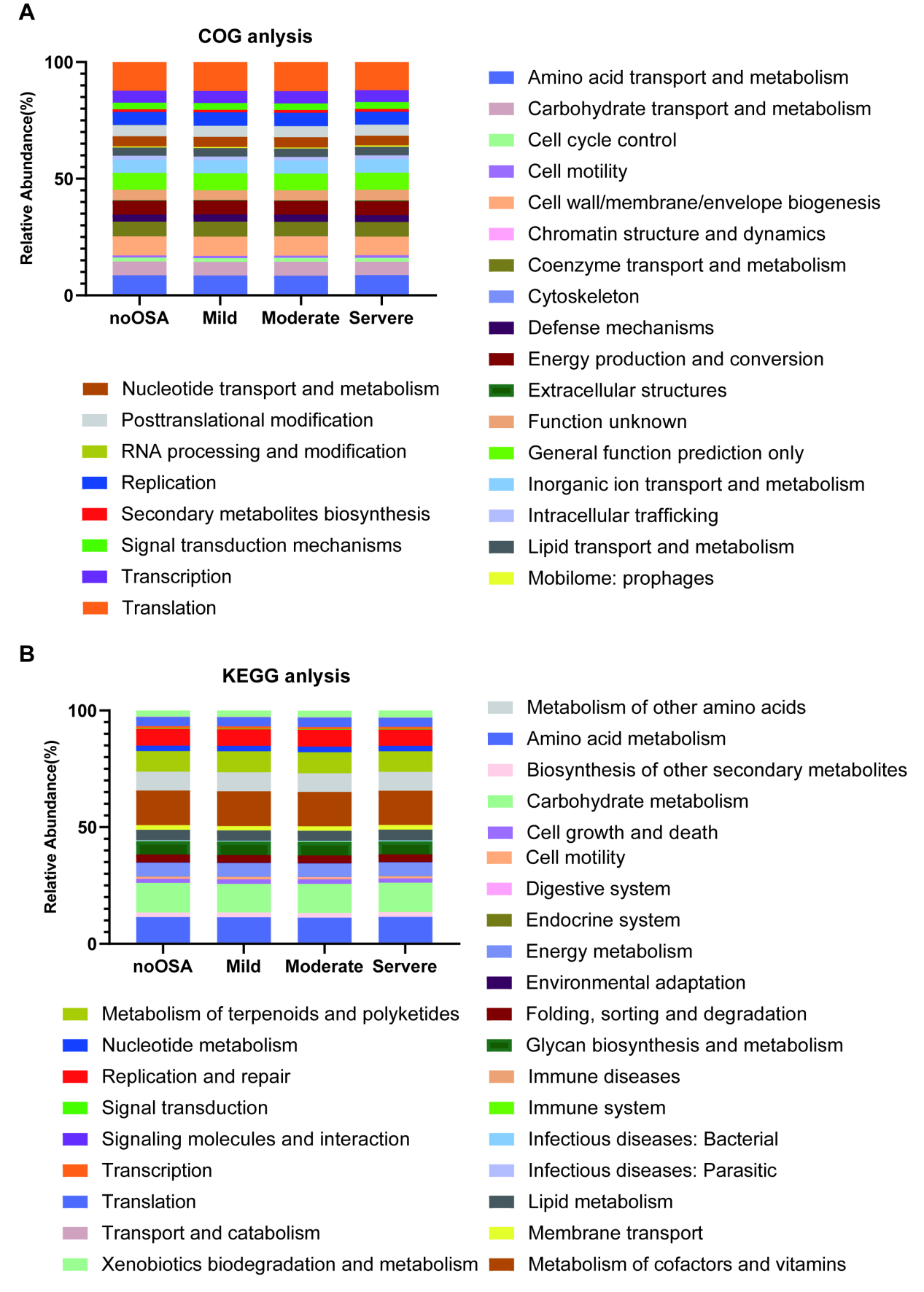


Figure S9


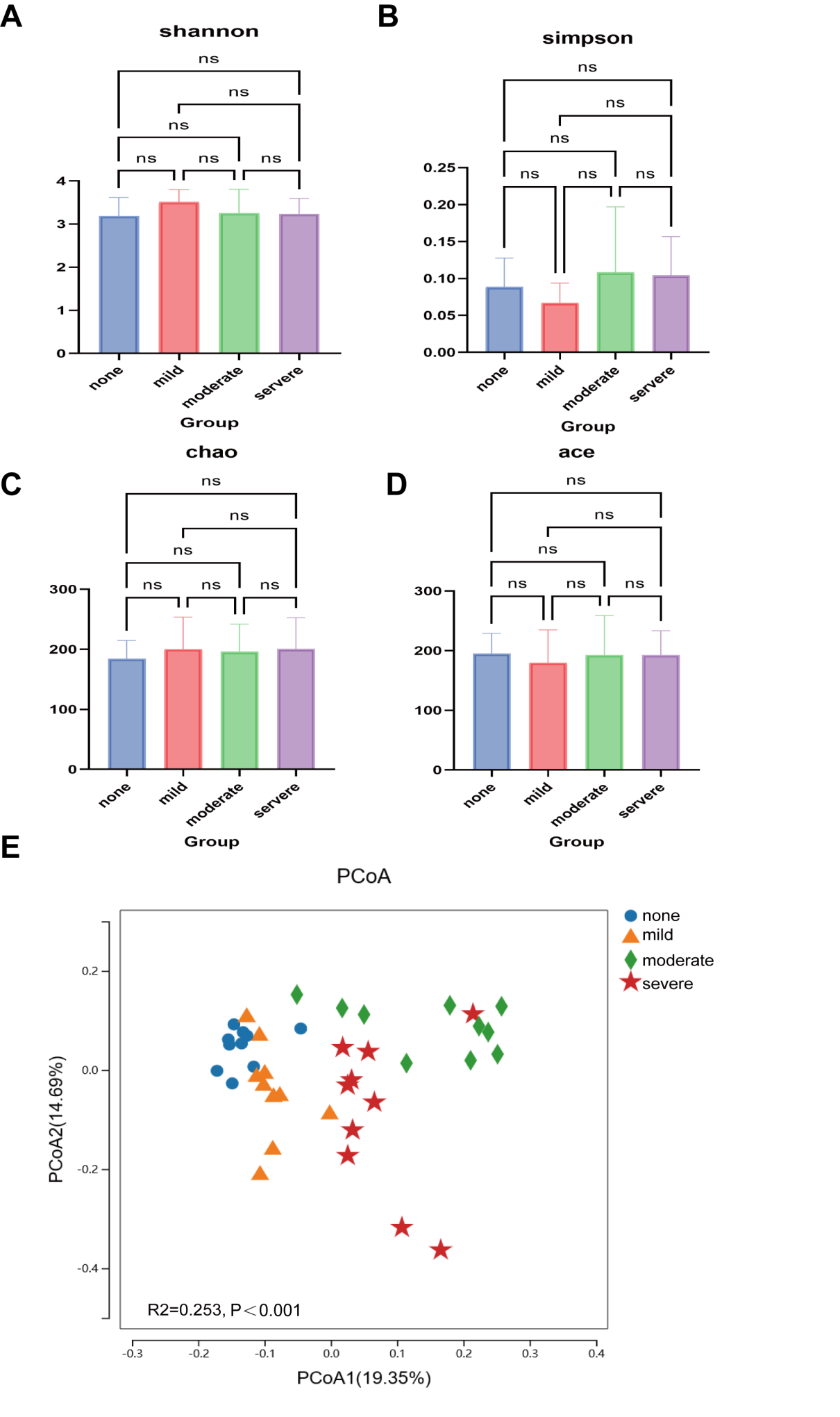


Figure S10


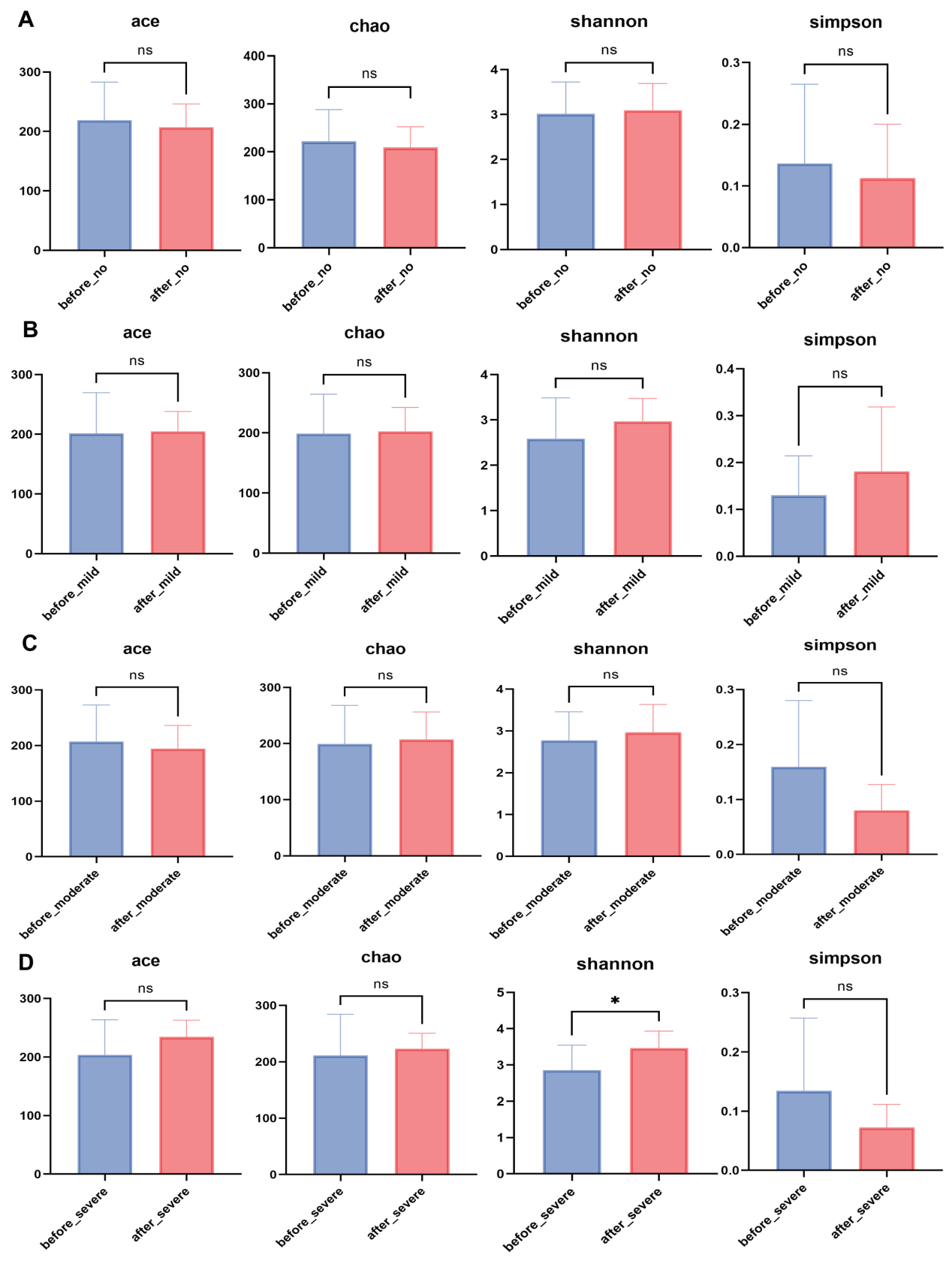


|  |  |  |  |  |  |  | |  |  |  |  |  |  |  |  |
| --- | --- | --- | --- | --- | --- | --- | --- | --- | --- | --- | --- | --- | --- | --- | --- |
|  |  |  |  |  |  | Tonsil recess | | |  |  |  |  |  |  |  |
|  | Prevotella | | Fusobacterium | | Veillonella | | Treponema | | | Neisseria | | Haemophilus | | Alloprevotella | |
|  | β1 | P-value | β2 | P-value | β3 | P-value | β4 | | P-value | β5 | P-value | β6 | P-value | β7 | P-value |
| Age | 0.09222 | 0.0041 | 0.09466 | 0.0007 | 0.4601 | 0.0599 | -0.03789 | | 0.777 | 0.08507 | 0.5688 | 0.07966 | 0.0796 | 0.2532 | 0.1351 |
| BMI | 0.2792 | <0.0001 | 0.1724 | 0.0001 | 0.3019 | 0.4096 | 0.1765 | | 0.3942 | 0.3283 | 0.1594 | 0.2381 | 0.0015 | 0.4919 | 0.0623 |
| OAHI | 0.1917 | 0.0766 | 0.02694 | 0.7592 | -1.161 | 0.1723 | -0.1755 | | 0.7108 | 1.168 | 0.0336 | 0.1053 | 0.5016 | 0.4775 | 0.4186 |
| MAI | 0.1944 | 0.0003 | 0.1176 | 0.0051 | -0.2846 | 0.445 | 0.0272 | | 0.8967 | 0.4974 | 0.0405 | 0.1564 | 0.0305 | 0.3525 | 0.1826 |
| MBOS | 1.189 | <0.0001 | 1.177 | <0.0001 | 2.905 | 0.0052 | 1.326 | | 0.0207 | 1.43 | 0.0246 | 1.245 | <0.0001 | 1.722 | 0.016 |
| WBCC | 0.08081 | <0.0001 | 0.07293 | <0.0001 | 0.08851 | 0.4435 | 0.09099 | | 0.1688 | 0.2007 | 0.0093 | 0.04945 | 0.0276 | 0.1514 | 0.0687 |
|  |  |  |  |  |  |  |  | |  |  |  |  |  |  |  |
|  |  |  |  |  |  |  |  | |  |  |  |  |  |  |  |
|  |  |  |  |  | Tonsillar capsule after tonsillectomy | | | |  |  |  |  |  |  |  |
|  | Prevotella | | Fusobacterium | | Veillonella | | Treponema | | | Neisseria | | Haemophilus | | Alloprevotella | |
|  | β1 | P-value | β2 | P-value | β3 | P-value | β4 | | P-value | β5 | P-value | β6 | P-value | β7 | P-value |
| Age | 0.1772 | 0.0094 | 0.06622 | 0.5066 | 0.1293 | 0.3245 | -0.2087 | | 0.3693 | -0.0203 | 0.9143 | 0.1366 | 0.0239 | 0.5144 | 0.0781 |
| BMI | 0.3968 | 0.0003 | 0.03356 | 0.8236 | 0.1162 | 0.5566 | 0.1617 | | 0.6442 | 0.7812 | 0.0102 | 0.2878 | 0.0025 | 0.7362 | 0.0951 |
| OAHI | 0.2951 | 0.143 | -0.1407 | 0.6468 | -0.1857 | 0.6443 | -0.04577 | | 0.9487 | 1.472 | 0.0166 | -0.08278 | 0.6422 | -0.02671 | 0.9756 |
| MAI | 0.1987 | 0.0733 | 0.1396 | 0.4063 | 0.2914 | 0.1901 | 0.01422 | | 0.9708 | 0.5964 | 0.0685 | 0.005075 | 0.9582 | 0.6425 | 0.1852 |
| MBOS | 1.941 | <0.0001 | 0.8581 | 0.1242 | 1.415 | 0.0559 | 0.1772 | | 0.8889 | 2.513 | 0.0212 | 1.239 | 0.0005 | 5.412 | 0.0016 |
| WBCC | 0.1181 | 0.0007 | 0.04052 | 0.4022 | 0.1789 | 0.0079 | 0.01506 | | 0.8927 | 0.1715 | 0.0686 | 0.05908 | 0.0416 | 0.3432 | 0.0178 |

**Table S1.** Multivariate linear regression analysis between clinical characteristics and microbiota signatures in the tonsil area.

**Table S2.** Functional pathway difference analysis

|  |  |  |  |
| --- | --- | --- | --- |
| **COG** |  |  |  |
| **Mild OSA vs. Non-OSA** | **OSA_mild(%)** | **Non_OSA(%)** | **P value** |
| Cell cycle control, cell division, chromosome partitioning | 1.597591 | 1.631424 | 0.009229 |
| Cytoskeleton | 0.012231 | 0.00798 | 0.010637 |
| RNA processing and modification | 0.011231 | 0.014509 | 0.034588 |
| **Moderate OSA vs. No OSA** | **OSA_moderate(%)** | **No_OSA(%)** | **P value** |
| Extracellular structures | 0.233611 | 0.28652 | 0.029474 |
| **Severe OSA vs. No OSA** | **OSA_severe(%)** | **No_OSA(%)** | **P value** |
| Mobilome: prophages, transposons | 0.603021 | 0.497117 | 0.016538 |
| Cell motility | 1.066121 | 0.972981 | 0.0336 |
| Extracellular structures | 0.356745 | 0.28652 | 0.035538 |
| **KEGG** |  |  |  |
| **Mild OSA vs. Non-OSA** | **OSA_mild(%)** | **Non_OSA(%)** | **P value** |
| Staphylococcus aureus infection | 0.021336 | 0.038786 | 0.004258 |
| Penicillin and cephalosporin biosynthesis | 0.021219 | 0.030472 | 0.008345 |
| C5-Branched dibasic acid metabolism | 1.38406 | 1.533965 | 0.009771 |
| Phosphotransferase system (PTS) | 0.309131 | 0.380352 | 0.01885 |
| Benzoate degradation | 0.157896 | 0.142704 | 0.019865 |
| Glycerophospholipid metabolism | 0.617319 | 0.664346 | 0.020929 |
| Terpenoid backbone biosynthesis | 1.565123 | 1.517479 | 0.027707 |
| Sulfur metabolism | 0.815414 | 0.889194 | 0.029125 |
| Protein processing in endoplasmic reticulum | 0.046494 | 0.040474 | 0.032148 |
| Carotenoid biosynthesis | 0.01073 | 0.017102 | 0.033757 |
| Plant-pathogen interaction | 0.196788 | 0.186454 | 0.035436 |
| Vitamin B6 metabolism | 1.248855 | 1.277262 | 0.037184 |
| Cell cycle - Caulobacter | 1.782581 | 1.741411 | 0.043895 |
| **Moderate OSA vs. Non-OSA** | **OSA_moderate(%)** | **Non_OSA(%)** | **P value** |
| Naphthalene degradation | 0.107569 | 0.028162 | 0.007944 |
| C5-Branched dibasic acid metabolism | 1.369108 | 1.533965 | 0.010391 |
| Systemic lupus erythematosus | 0.003878 | 0.007836 | 0.013253 |
| Carotenoid biosynthesis | 0.009692 | 0.017102 | 0.020349 |
| Pyrimidine metabolism | 1.373729 | 1.343396 | 0.039124 |
| Fructose and mannose metabolism | 0.952768 | 0.91861 | 0.048016 |
| Methane metabolism | 0.439285 | 0.452869 | 0.049652 |
| **Severe OSA vs. Non-OSA** | **OSA_severe(%)** | **Non_OSA(%)** | **P value** |
| Phosphonate and phosphinate metabolism | 0.170292 | 0.149847 | 0.010604 |
| Amino sugar and nucleotide sugar metabolism | 1.025317 | 1.063392 | 0.015543 |
| Tyrosine metabolism | 0.32856 | 0.298149 | 0.023077 |
| Carotenoid biosynthesis | 0.027606 | 0.017102 | 0.024481 |
| Drug metabolism - other enzymes | 1.020101 | 1.212715 | 0.030045 |
| Ethylbenzene degradation | 0.102285 | 0.03999 | 0.030123 |
| Glutathione metabolism | 0.781231 | 0.722728 | 0.0336 |
| Tryptophan metabolism | 0.235696 | 0.199296 | 0.036036 |
